# Characterising the Influence of Genetics on Breast Muscle Myopathies in Broiler Chickens

**DOI:** 10.3389/fphys.2020.01041

**Published:** 2020-08-20

**Authors:** Richard A. Bailey, Eduardo Souza, Santiago Avendano

**Affiliations:** ^1^Aviagen, Newbridge, United Kingdom; ^2^Aviagen, Huntsville, AL, United States

**Keywords:** broiler, breast muscle, meat quality, myopathy, heritability, genetics, breeding

## Abstract

This report provides the first estimates of the genetic basis of all key breast muscle myopathies (BMM) in broiler chickens [Deep pectoral myopathy, wooden breast, white striping and spaghetti breast] and their relationship with body weight and breast yield. Data from a pure bred high yielding commercial broiler line were analysed to estimate the genetic parameters using a multivariate animal model with the appropriate fixed effects and permanent environmental effect of the dam. Heritabilities of the BMM ranged from 0.04 to 0.25 and the genetic correlation of the BMM with body weight and breast yield ranged from -0.06 to 0.41. Here we highlight that the genetic variance of BMM accounts for a low proportion of the phenotypic variance and the BMM have a low genetic relationship with performance traits. The large contribution of residual variance to the phenotypic variance for the BBM was >71.5% which indicates the importance of the non-genetic effects on BMM. The data presented also show that the moderate to low genetic influence for the development of BMM can be used, through balanced selection, to reduce the myopathy incidence in the long term. The impact of genetic selection against BMM was tested empirically by comparing the incidence of WB and % breast yield of a commercial broiler with a high generation (HG) broiler. The HG broiler used represents 2 years of genetic improvement compared to the commercial broiler; the HG broiler had an 18.4% relative decrease in WB and a 1.02% relative increase in breast yield compared to the commercial broiler. This paper describes the relationship between the genetic and non-genetic factors influencing BMM highlighting the importance of understanding the non-genetic effects on myopathy incidence. It also shows that the genetic component of BMM can be reduced whilst at the same time improving breast yield as part of balanced breeding goals.

## Introduction

Poultry production is one of the most sustainable and efficient systems for producing high quality affordable animal protein for human consumption. The increasing efficiency of poultry production is the result of advances in livestock production systems, nutrition, animal breeding and genetic selection. Significant improvements in breast meat yield, live weights and biological efficiency have been proved to be achieved through genetic selection (Havenstein et al., 2003a, b; Fleming et al., 2007; Mussini, 2012; Zuidhof et al., 2014). While it is possible that selection for production traits can have undesirable consequence on bird fitness (Hocking, 2014), these negative consequences are mitigated through balanced selection where health and welfare traits are included as equally important selection criteria (McKay et al., 2000; Neeteson, 2010; Dawkins and Layton, 2012; Avendaño et al., 2017). This is demonstrated by Kapell et al. (2012a, b) where they show that it has been possible to simultaneously improve both production traits and welfare related traits such as leg defects and contact dermatitis. Carcase yield and live weight are key performance traits for broiler chickens and as part of balanced breeding goals there is also a focus on carcase quality to reduce conditions such as ascites, infections, skeletal deformities and muscle myopathies. The impact of continued improvement of carcase quality, along with improvements of live weight and carcase yield, is evident in data from the United States poultry industry where carcase condemnations (combined *post-* and *ante mortem*) have decreased from 2.65% to 0.54% over the last 30 years (USDA, 2020). One cause of carcase downgrades or condemnation is breast muscle myopathies (BMM) which can result in economic losses to the poultry meat producers (Mitchell, 1999; Kuttappan et al., 2016; Barbut, 2019). One of the most widely recognised and well characterised myopathies is deep pectoral myopathy which was first reported in chickens in the 1980’s (Richardson et al., 1980). This myopathy is the result of ischaemic necrosis of the deep pectoral muscle (*Pectoralis minor*) as a consequence of a compartment syndrome following exertion (Siller et al., 1978). As blood flows into the deep pectoral muscle during exercise, the inelastic fascia surrounding the muscle causes the intramuscular pressure to increase which in turn impedes venous output from the muscle causing blood pooling (Siller and Wight, 1978; Richardson et al., 1980). The consequence of this ischaemia is rapid necrosis of the muscle tissue and erythrocytes which manifests as haemorrhaging followed by a greenish discolouration as the haemoglobin in the red blood cells is broken down (Bianchi et al., 2006). One of the key triggers for this myopathy is excessive flapping or excitability within the flock and clear management recommendations are available to control the field incidence of this condition (Siller et al., 1978; Bilgili and Hess, 2008; Lien et al., 2012).

**FIGURE 1:**
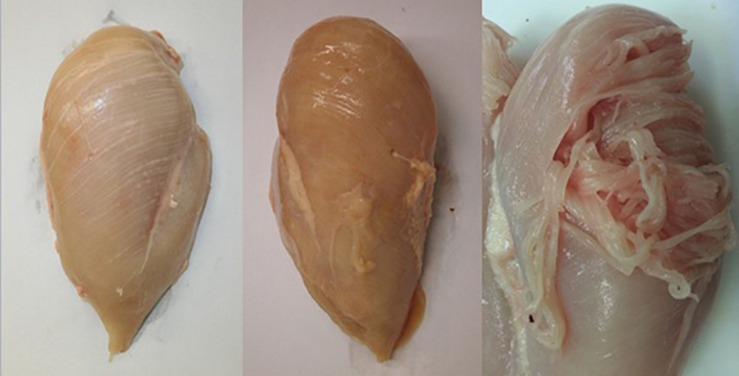
Breast fillets showing the novel myopathies white striping (WS; left), wooden breast (WB; middle), and spaghetti breast (SB; right).

Over the last decade there has been a renewed focus on BMM within the poultry industry following increased reports of novel myopathies affecting the *Pectoralis major* muscle in a number of commercial broiler strains (Kuttappan et al., 2012a, 2013a,c; Ferreira et al., 2014; Mudalal et al., 2015). Within the industry vernacular these myopathies are commonly referred to as ‘White striping’, ‘Wooden breast’ and ‘Spaghetti breast’ owing to their distinctive presentation on inspection (Figure 1; Kuttappan et al., 2013c; Sihvo et al., 2014; Baldi et al., 2018; Huang and Ahn, 2018; Barbut, 2019). White striping (WS) is characterised by white lines running in parallel to the muscle fibres on the surface of the muscle; there is bird to bird variation in the quantity and thickness of these lines which has enabled severity scoring when it comes to recording of this myopathy (Bailey et al., 2015; Kuttappan et al., 2016). These white lines have, through extensive histological and chemical analysis, been shown to be composed of adipose tissues (Kuttappan et al., 2013a; Bailey et al., 2015). Wooden Breast (WB) manifests as a hardening of the breast fillet which has been proposed to be either due to increased collagen crosslinking (Velleman and Clark, 2015) or due to increased water being trapped within the muscle myofibrillar space (Tasoniero et al., 2017). The hardening of the muscle can occur as a singular focal lesion or multiple focal lesions, and in more severe cases can affect the whole fillet (Sihvo et al., 2017; Huang and Ahn, 2018). Depending on the severity of the condition there can be additional macroscopic features such as a pale colour, surface haemorrhaging and the presence of a sterile transudate on the surface of the muscle. Histological analysis of affected muscles show ongoing degeneration with active regeneration of muscle fibres, infiltration of immune cells along with increased deposition of adipose and connective tissues (Sihvo et al., 2014; Velleman and Clark, 2015). Spaghetti breast (SB), so called because of its resemblance to spaghetti pasta (Baldi et al., 2018), is characterised by a loss of integrity of the muscle fibres in the breast muscle leading to friability and loosening of the muscle tissue (Barbut, 2019). As with the other myopathies the severity of SB is variable, ranging from only a small part of the breast muscle being affected to the whole muscle showing the condition. Histological analysis of SB reveals a disorganised distribution of large and small muscle fibres interspersed with diffuse connective tissue.

**FIGURE 2:**
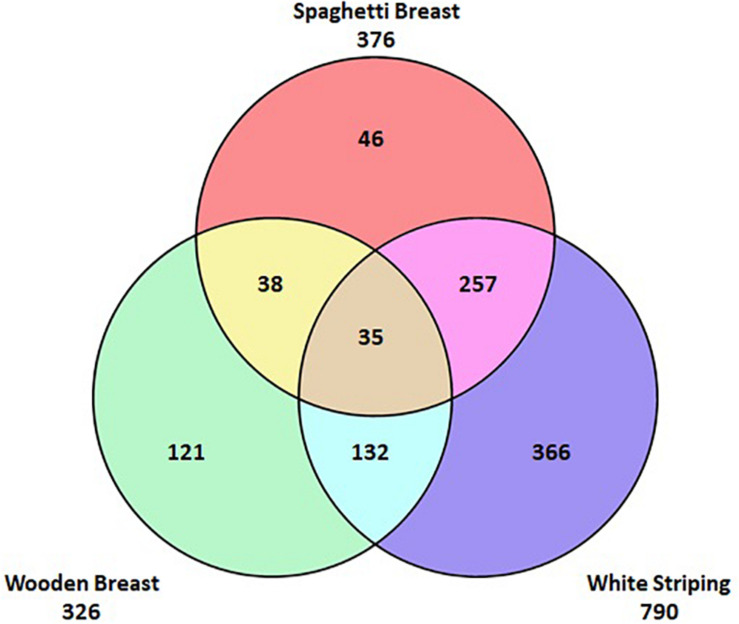
This venn diagram demonstrates the incidence of the myopathies in 995 myopathy affected fillets. It can be seen that the myopathies can occur individually or be comorbid with each other.

Unlike DPM, the aetiology of these novel myopathies is not fully understood nor is the relationship they have with each other. It has been suggested that they are a progression of the same condition and other opinions suggest that they are distinct conditions. Figure 2 shows the incidence of the myopathies in a batch of affected fillets; it can be seen that they can occur individually or in combination with each other. The concurrent occurrence of the myopathies must be taken into account when selecting fillets to investigate each of the myopathies to ensure that it is just one myopathy under investigation. Failure to do so could result in wrongly assigning a biological or biochemical feature of one myopathy to its comorbid counterpart. There have been a number of studies aiming to understand the pathogenesis of the myopathies through the use of gene expression along with proteomic and metabolomic analyses (Mutryn et al., 2015; Velleman and Clark, 2015; Zambonelli et al., 2016; Kuttappan et al., 2017; Boerboom et al., 2018; Cai et al., 2018). Results from gene expression studies show that muscles affected by the myopathies have an increased expression of a range of genes associated with metabolic (e.g. hypoxia, oxidative stress, calcium metabolism, fat metabolism, inflammation), anatomical, and structural biological processes (Mutryn et al., 2015; Zambonelli et al., 2016; Papah et al., 2018; Soglia et al., 2020). Comprehensive studies looking at birds of different ages and different locations in the muscle have highlighted early perturbations in affected muscle and localised changes in metabolic activity within the muscle which can be linked to the development of myopathies (Clark and Velleman, 2017; Papah et al., 2018; Brothers et al., 2019; Lake et al., 2019). Through proteomics, Kuttappan et al. (2017) reported that levels of proteins involved in cellular movement, carbohydrate metabolism, protein synthesis and protein maturation were significantly different in breast fillets with WB compared to breast muscles without WB. Boerboom et al. (2018) identified biological pathways which may explain how WS manifests; they reported significant differences in carbohydrate metabolism and fatty acid composition of the WS fillets compared to unaffected fillets. In addition they confirmed the findings of the gene expression studies reporting evidence of hypoxia and oxidative stress within the affected fillets. Both hypoxia and oxidative stress have been proposed as causes of the histological abnormalities seen in affected muscle tissue (Sihvo et al., 2014; Velleman and Clark, 2015) which could be linked to discord between muscle fibre size and vascular supply leading to a compartment syndrome (Lilburn et al., 2019). Overall, these studies indicate that there are differences in a range of metabolic, anatomical and structural biological processes within the muscle of affected birds when compared to unaffected breast muscles. These findings are invaluable as they help to reveal the underlying biology and ongoing metabolic processes allowing for the development of biomarkers for further mapping of the pathophysiology of the myopathies. However, whilst these studies inform on the differences in biology between the breast muscles of affected and unaffected birds it is still unclear as to what is causing the initial disruption within the tissues. It is an attractive option to speculate about a potential singular causes for the myopathies based on the outcome of these studies alone; however, there is a need to understand what factors trigger the onset of these conditions leading to the biological changes detected in the studies mentioned above.

A popular theory for the cause of the myopathies is the genetic selection for increased body weight and breast yield (Sihvo et al., 2014; Papah et al., 2018; Petracci et al., 2019; Soglia et al., 2019); to date there is limited information on the genetic basis of the myopathies in broiler populations and their correlations with each other and production traits. A previous analysis of two pure bred commercial broiler genotypes showed low to moderate heritability for WB (0.024–0.097) and WS (0.185–0.338) indicating that whilst there is a genetic component to the myopathies the non-genetic factors play a much greater role in the manifestation of WB and WS (Bailey et al., 2015). In both broiler populations analysed, WB and WS both had modest genetic correlations with body weight and breast yield (-0.027–0.248). This indicates that selection for body weight and breast yield alone do not explain the manifestation of the myopathies and more research is needed to fully understand how the myopathies initiate and develop. A further study by Alnahhas et al. (2016) showed a higher estimate of heritability of 0.65 for WS in divergently selected broiler lines. However, as the authors rightly described, they measured WS on an underlying continuous scale which can result in a higher heritability estimate compared to the 4 point scale used by Bailey et al. (2015). According to data published by Dempster and Lerner (1950), a heritability of 0.65 would correspond to a heritability of 0.41 on the observed categorical scale which would bring it closer to the heritability for WS described by Bailey et al. (2015). A further difference in these two studies is the fitting of the effect of the common maternal environment; Alnahhas et al. (2016) did not fit this effect to WS therefore this effect would overestimate the genetic variance and heritability estimate in their analysis. Conversely, Bailey et al. (2015) accounted for the common maternal environment effect contributing to the total variance of the myopathies therefore allowing a full discrimination from the additive genetic variance. To date there is no information published on the genetic basis of SB; the aim of this study is to extend the work by Bailey et al. (2015) to further characterise the genetic basis of BMM by estimating heritabilities for DPM, WB, WS and SB along with their genetic correlations with body weight and breast yield. In addition, we will empirically illustrate the impact of genetic selection for reducing WB whilst continuing to improve breast yield as part of a balanced breeding goal.

## Materials and Methods

### Study 1: Estimation of Genetic Parameters of Breast Muscle Myopathies

#### Birds, Housing and Management

The data analysed in this study comes from the routine meat quality traits recording on a pure bred commercial broiler line from within the Aviagen (Tennessee, United States) breeding program. The phenotypic data spans four generations collected over 3 years from 105 flocks, with the inclusion of an extra generation of pedigree relationships for the estimation of the genetic parameters. Within this study the key phenotypic traits of interest were broiler body weight (BW), breast meat yield (BY), deep pectoral myopathy (DPM), WB, SB and WS (Table 1). The birds were housed within environmentally controlled pedigree broiler farms in Crossville Tennessee, United States; a detailed description of environmental parameters can be found in Table 2. The birds were all housed in pens with wood shavings provided as the litter substrate with *ad libitum* access to food and water. All birds were incubated in the same hatchery with the same incubation conditions, after hatch all birds received the required vaccinations and were tagged with a unique barcoded wingband for identification.

**TABLE 1:** Number of records for each trait used in the analysis for the estimation of genetic parameters in the pure line broiler population in study 1.

Trait	Number of records
Body weight (BW)	154781
Breast yield (BY)	34794
Deep pectoral myopathy (DPM)	35394
Wooden breast (WB)	38730
Spaghetti breast (SB)	38773
White striping (WS)	38780

**TABLE 2:** Environmental parameters for all farms with birds under examination for both studies reported in this paper.

Environmental parameter	Target
Feed days: 0–10	Starter (259 *g* CP/kg; 12.34 MJ ME/kg)
Feed days: 11–25	Grower (234 *g* CP/kg; 12.84 MJ ME/kg)
Feed days: 25-final weighing	Finisher (212 *g* CP/kg; 13.04 MJ ME/kg)
Stocking density	34.1 to 36 kg bird weight per m2
Temperature	Gradually reduced from 29 to 20°C
Photoperiod day 0–7	23L:1D
Photoperiod day 8-final weighing	20L:4D
Light intensity day 0–7	40 lux
Light intensity day 8-final weighing	Gradually reduced from 20 to 10 lux

#### Recording of Traits

All birds were given a uniquely barcoded wingband after hatch and all traits measured were recorded to this number. The birds were individually weighed at 40 days of age and then taken to the processing plant. Following plucking and evisceration, the carcasses were air chilled with a target to reach 3°C within 8–10 h of kill. the carcases were deboned 24 h after kill and the presence of BMM was then assessed and recorded by a trained team of individuals as previously described in Bailey et al. (2015). DPM was recorded as present or absent, whereas WB, SB and WS were all scored on a severity scale. WB severity was based on a four point scale, where score 0 is normal, score 1 is a minor focal lesion of WB, score 2 is multiple focal lesions of WB and score 3 is extensive WB across the whole muscle. SB was scored on a three point scale where score 0 is normal, score 1 is less than 50% of the breast affected and a score 2 is more than 50% of the breast affected by SB Similarly, WS was recorded on a 4 point scale of severity; a score of 0 represents no striping, score 1 is noticeable striping covering only part of the breast, score 2 is a breast fillet with extensive striping covering the breast surface and score 3 is extensive coverage with very thick stripes. The scoring criteria for each BMM capture the range of mild to severe phenotypes which were then used for the estimation of genetic parameters.

#### Statistical Analyses

The traits BW, PW, BY, DPM, WB, SB and WS were analysed together in the following multivariate animal model to estimate genetic parameters:

y=X⁢b+Z⁢a+W⁢c+e,

Where: **y** denotes the vector of observations of the traits, **b** is the vector of the fixed effects accounting for the interaction between hatch-week, pen and contributing mating group of each flock. The vector of additive genetic effects is shown by **a**, the vector of permanent environmental effects of the dam (PEM) is represented by **c,** and **e** denotes the vector of residuals. **X**, **Z** and **W** represent incidence matrices relating the vectors **b**, **a**, and **c** to **y**. The assumed (co)variance structure was:

$$\text{V}\,\left[\begin{array}{c} \text{a}\\ \text{c}\\ \text{e} \end{array}\right]=\left[\begin{array}{ccc} \text{A}⊗\text{G} & 0 & 0\\ 0 & \text{I}⊗\text{C} & 0\\ 0 & 0 & \text{I}⊗\text{R} \end{array}\right]$$

Where: **A** represents the additive and genetic relationship matrix and **I** represents identity matrix. The variance and covariance matrices of additive genetic effects, PEM and residual effects are denoted by **G**, **C** and **R,** respectively. All variance component analyses were performed by REML using VCE (Groeneveld et al., 2008).

### Study 2: Empirical Testing of Genetic Selection to Reduce Myopathies

#### Birds, Housing and Management

**FIGURE 3:**
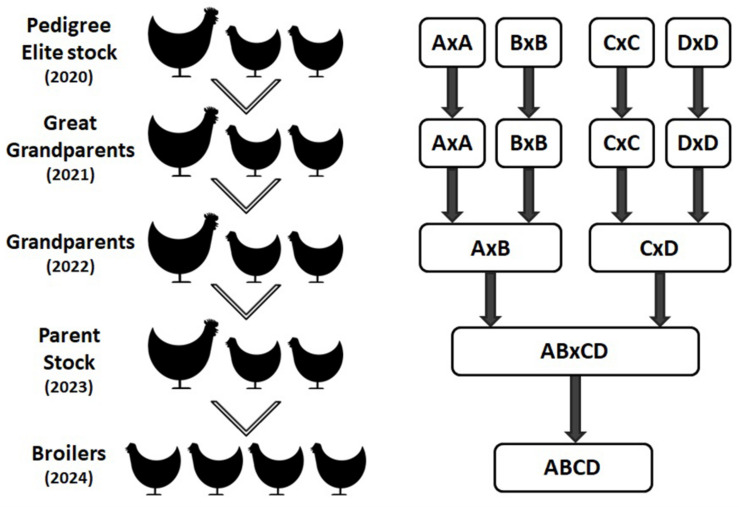
The commercial broiler is a four way cross of pure broiler lines (represented by A, B, C, and D) as demonstrated in this image. The movement of genetic material from the pedigree pure lines to the commercial broiler population takes around 4 years through great grandparent, grandparent, and parent multiplier flocks, i.e. the impact of any genetic selection made in 2020 will reach the broiler population in 2024.

**FIGURE 4:**
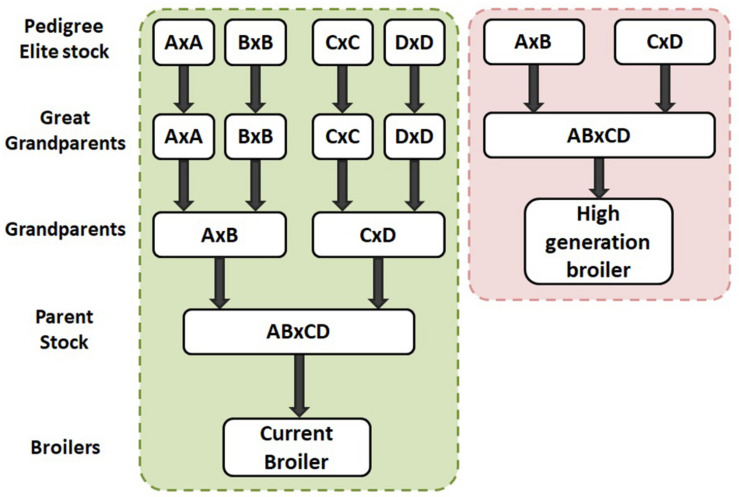
In study 2 a high generation broiler was produced by using great grandparents as parent stock by crossing pedigree pure lines (right dashed box). The high generation broiler can then be compared to the current commercial broiler (left dashed box) to see the impact of genetic selection for a trait of interest such as wooden breast. The generational difference between the two broiler groups represents 2 years of genetic progress.

The commercial broiler chicken is the result of a four way cross of pure bred broiler lines. The crossing of lines takes place over multiple generations requiring four parental generations, namely: Pedigree Elite Stock, Great grandparents (GGP), Great parents (GP) and Parent Stock (PS) as shown in Figure 3. Hence, it takes at least 4 years for the impact of genetic selection to reach the commercial broiler from the elite pedigree population. As part of Aviagen’s ongoing strategy to reduce the genetic propensity for the expression of BMM every selection candidate of all lines is assessed and scored on farm for WB through palpation, while their siblings are assessed and scored for the presence of all BMM through carcase evaluation following processing. This data is then used to select against individuals with a higher propensity for the BMM. A higher propensity for BMM is based on predicted breeding values; a higher propensity for BMM would be considered those individuals which have a higher than average breeding value for BMM. In order to empirically demonstrate the impact of this genetic selection we generated a ‘high generation’ (HG) broiler by fast tracking the generation of PS from the mating of birds from the GGP generation as described in Figure 4. The impact of genetic selection against the genetic propensity to WB can be then assessed through comparing the performance of the HG and the current commercial broiler. In this study, hatching eggs for commercial Ross 708 broilers were incubated alongside hatching eggs for HG Ross 708 broilers as per the Aviagen recommendations^1^. The chicks of both groups were reared side by side in separate pens as commercial broilers as per the Aviagen Ross 708 broiler handbook^1^; A total of four consecutives hatches were placed and a sample of the birds of each group, commercial and HG broiler, were sent for processing (Table 3). Hatching eggs for the HG broilers originated from the same parent flock for each of the four consecutive hatches whereas hatching eggs for commercial broilers were obtained from commercial field flocks which were all age matched where possible (Table 3). PS for both commercial and HG broilers were fed feed from the same feed mill and the same management parameters were used during rear and lay as per the Aviagen PS management guide.

**TABLE 3:** Placement date, parent stock age, broiler age and average (AVG) bodyweight per hatch and number of birds processed and assessed for wooden breast myopathy per group in study 2 comparing commercial Ross 708 broilers and high generation Ross 708 broilers.

Hatch^1^	Chick placement date	Age of parent flocks (weeks)^2^		Number of birds placed per group	Age of processing (days)	AVG Body weight (g)	Number of birds processed per group
1 (M+F)	21st March	38		600	55	4337	160
2 (M+F)	18th April	41		640	62	4600	80
3 (M+F)	30th May	48		480	62	4300	80

4 (only M)	11th July	HG 54	Com 39	150	55	4331	120

***^1^**Within each hatch there were an equal number of birds in each group of broiler line. In hatch 1, 2, and 3 there was an equal split of males and females placed. ^2^For hatches 1–3 the parent age was the same for both groups of broilers. In hatch 4 the parent ages were different for the high generation (HG) broiler and the current commercial (Com) broiler.*

#### Recording of Traits

Birds were processed as outlined in the previous section above. Breast meat was weighed and recorded as a % of body weight to give breast yield. All breast fillets were scored for WB on the same 4 point scale described above.

#### Data Analysis

Due to hatch to hatch variation in performance, as shown by the mean BW in Table 3, the data for breast yield and myopathy incidence were normalised to relative values in order to visualise and compare the impact of genetic selection across all four hatches of birds in study 2. This was performed by the following formula:

$$\begin{array}{c} R\,e\,l\,a\,t\,i\,v\,e\,v\,a\,l\,u\,e=1-\\ \frac{\left(T\,r\,a\,i\,t\,v\,a\,l\,u\,e\,s\,f\,o\,r\,c\,u\,r\,r\,e\,n\,t\,b\,r\,o\,i\,l\,e\,r-T\,r\,a\,i\,t\,v\,a\,l\,u\,e\,f\,o\,r\,h\,i\,g\,h\,g\,e\,n\,e\,r\,a\,t\,i\,o\,n\,b\,r\,o\,i\,l\,e\,r\right)}{T\,r\,a\,i\,t\,v\,a\,l\,u\,e\,o\,f\,t\,h\,e\,c\,u\,r\,r\,e\,n\,t\,b\,r\,o\,i\,l\,e\,r} \end{array}$$

Any significance of differences between the two broiler groups in each hatch were determined with a student’s *t*-test.

## Results

### Study 1: Estimation of Genetic Parameters of Breast Muscle Myopathies

The summary of production traits and the mean scores of BMM for the pure line population can be seen in Table 4. The mean BW was 3.03 kg and the mean BY was 28.9%, respectively. For the BMM: WB and WS had mean scores of 0.30 and 0.19, respectively; both DPM and SB had lower mean scores of 0.16 and 0.04.

**TABLE 4:** Descriptive statistics for the traits used in study 1 for the estimation of genetic parameters in the pure broiler line.

Trait	Mean	SD
**Body weight Kg (BW)**	3.03	0.37
**% Breast Yield (BY)^1^**	28.9	2.30
**Deep pectoral myopathy (DPM)^2^**	0.15	0.11
**Wooden breast (WB)^2^**	0.19	0.10
**Spaghetti breast (SB)^2^**	0.04	0.03
**White striping (WS)^2^**	0.30	0.11

*^1^Breast yield is expressed as a percentage of body weight. ^2^The myopathies show the mean score based on the severity score mentioned in the materials and methods section.*

Table 5 displays the heritabilities of the traits analysed in study 1 along with the genetic and phenotypic correlations between the traits. The measures of genetic and phenotypic correlations range from -1 (an antagonistic relationship) to 1 (a positive relationship) and it describes any shared genetic background or phenotypic relationship between two traits.

**TABLE 5:** Genetic parameter results for study 1.

BW	BY	DPM	WB	SB	WS
**0.31_(0.03)_**	−0.06_(0.02)_	0.14_(0.03)_	0.20_(0.04)_	−0.06_(0.03)_	0.23(_0.04)_
0.15	**0.40_(0.02)_**	0.23_(0.02)_	0.41_(0.03)_	0.36_(0.02)_	0.31_(0.01)_
0.03	0.02	**0.06_(0.02)_**	0.46_(0.02)_	0.04_(0.03)_	0.34_(0.03)_
0.08	0.13	0.10	**0.07_(0.04)_**	−0.04_(0.02)_	0.74_(0.04)_
0.02	0.17	0.03	−0.02	**0.04_(0.02)_**	0.02_(0.01)_
0.23	0.22	0.12	0.25	0.05	**0.25_(0.02)_**

*Estimates of heritabilities (bold, diagonal), genetic correlations (above diagonal), and phenotypic correlations (below diagonal) for body weight (BW), breast yield (BY), deep pectoral myopathy (DPM), wooden breast (WB), spaghetti breast (SB) and white striping (WS). Standard errors are displayed in parentheses.*

The phenotypic correlations between the BMM and the production traits BY and, BW were low to moderate ranging from 0.02 to 0.23; this indicates the BMM are not linked wholly to changes in BW or BY. Equally the phenotypic correlations between the individual myopathies were generally low to moderate ranging from -0.02 to 0.25; thus suggesting the individual BMM are independent of each other. The genetic correlation between BY with BW was low with an estimate of -0.06 indicating these two traits are independent of each other. The genetic correlations between the BMM and BW and BY were low to moderate with estimates ranging from -0.06 to 0.41. This indicates there is limited shared genetic background between BW and BY with the BMM. The genetic correlations between the myopathies were predominately low to moderate ranging from -0.04 to 0.52 with a higher genetic correlation between WB and WS of 0.74.

Heritability is the proportion of phenotypic variance explained by genetic variance, and thus provides an indication of the genetic influence on variance on any given trait. The estimated heritabilities for BW and BY were 0.31 and 0.40, respectively, and the estimated heritabilities for the BMM ranged from low to moderate: 0.04 for SB, 0.06 for DPM, 0.07 for WB and 0.25 for WS. Table 6 shows the proportion of phenotypic variance that was accounted for by the environmental and maternal environment effects. For BW and BY the PEM effect accounted for 0.5 and 1.0% of the phenotypic variance, respectively, thus accounting for only a very small proportion of the phenotypic variance. For the BMM the PEM similarly accounted for a small proportion of the phenotypic variance ranging from 0.3 to 3.4%. Overall, the results show that the majority of the phenotypic variance of BMM traits analysed in study 1 is explained by the environmental (i.e. residual) variance. The residual variance for BMM accounts for the larger proportion of the phenotypic variance with a range of 71.5–95.2%. Thus, these results indicate a modest genetic influence on the variation in BMM at 40 days in the line studied; however, there is a much greater variation resulting from the non-genetic factors.

**TABLE 6:** Phenotypic (PHEN), residual (RES), maternal permanent environmental (PEm) variances and proportions of phenotypic variance accounted for by RES (Prop RES) and PEm (Prop PEm) for body weight (BW), breast yield (BY), deep pectoral myopathy (DPM), wooden breast (WB), spaghetti breast (SB) and white striping (WS) analysed in study 1.

Trait	PHEN	RES	PEM	Prop RES	Prop PEm
BW	582.11	393.01	5.93	0.675_(0.007)_	0.010_(0.001)_
BY	3.06	1.82	0.01	0.595_(0.010)_	0.005_(0.003)_
WB	372.12	335.28	11.16	0.901_(0.004)_	0.030_(0.002)_
SB	166.42	158.50	1.14	0.952_(0.003)_	0.007_(0.002)_
WS	605.63	432.91	20.84	0.715_(0.005)_	0.034_(0.001)_
DPM	1154.06	1079.49	3.68	0.935_(0.004)_	0.003_(0.003)_

*Standard errors for PropRES and PropPEm are displayed in parentheses.*

### Study 2: Empirical Testing of Genetic Selection to Reduce Myopathies

**FIGURE 5:**
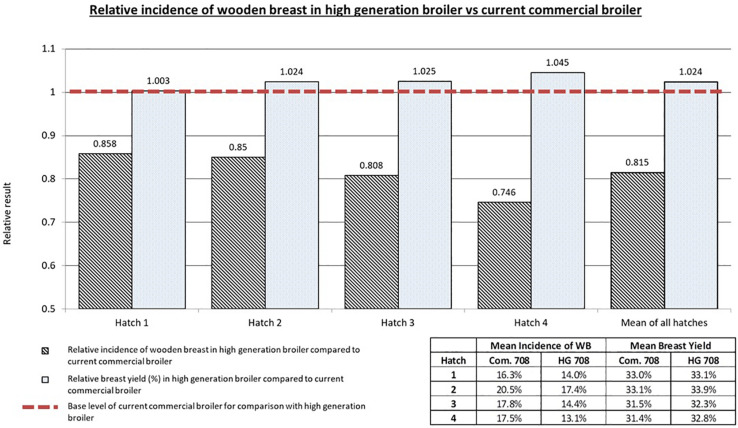
Relative results for wooden breast incidence (scores 1, 2 and 3) and breast yield of the high generation broiler compared to the current commercial broiler in study 2. Mean WB incidence and BY values are also given for all groups. An overall decrease in total wooden breast incidence can be seen as a result of genetic selection whilst simultaneously improvements in breast yield can be achieved.

The relative and mean BY and WB incidences for the HG and current commercial broilers across the four hatches are displayed in Figure 5. In each hatch a reduction in the relative incidence of WB can be seen in the HG broiler compared to the current commercial broiler. Simultaneously, in each hatch there is a relative increase in BY seen in the HG broiler compared to the current commercial broiler. Overall the higher generation broilers had on average 18.4% less WB (*p* < 0.001) and 2.4% more breast yield (*p* < 0.03) relative to the current commercial broiler. These results demonstrate that selection for increased breast meat in broilers can be achieved whilst selecting against the genetic propensity to develop BMM such as WB. The generational difference of the commercial and HG broilers used in this study represents 2 years. This means an annual realised relative reduction in WB of 9.2% and an annual realised relative increase in BY of 1.2%.

## Discussion

The importance of BMM in the poultry industry has been well documented in academic and popular poultry press over the last 10 years. Myopathies can lead to carcase downgrades in severe cases or, in the milder cases they can have a negative influence on the eating quality, ability of meat to take up marinates, cooking losses and consumer acceptance (Kuttappan et al., 2012b; Aguirre et al., 2016; Tijare et al., 2016; Bowker et al., 2018). A recent publication by Gratta et al. (2019) showed that, when compared to unaffected breast fillets, the microbial shelf life of breast fillets with WS or WB was significantly longer. This supports the general consensus that breast fillets affected by the myopathies do not pose a human health concern (Kuttappan et al., 2012b, 2013b). The aetiology of the breast myopathies is still unknown, however, various hypotheses have been proposed; a common hypothesis for the increased incidence of myopathies such as WB, WS and SB in broiler chickens is that they are an unexpected consequence of selection for body weight and breast yield (Zambonelli et al., 2016; Baldi et al., 2018; Petracci et al., 2019). Previous published data have estimated heritabilities and genetic correlations for WS individually (Alnahhas et al., 2016) and WB and WS together with DPM (Bailey et al., 2015). To the knowledge of the authors there are no data published on the genetic basis of SB; thus here we report the first dataset describing the genetic basis of SB. Furthermore, the analysis presented here is the first report characterising the genetic basis of all the BMM together and their relationship with bodyweight and breast yield in broiler chickens. The data presented here show a lower contribution of the BMM genetic variance to the phenotypic variance resulting in low heritability estimates and a large contribution of residual variance. This means, whilst there is a modest genetic component to the variation in myopathies, it is the non-genetic effects that are of greater importance which is in agreement with previous published results (Bailey et al., 2015).

In this study, BW and BY showed estimates of heritability of 0.31 and 0.40, respectively, which are similar in magnitude to those found in the literature (Bailey et al., 2015; Alnahhas et al., 2016). The heritabilities for DPM, WB and SB are low with estimates ranging from 0.04 to 0.07 indicating a very strong influence of the non-genetic factors in the variation in manifestation of these myopathies. It is well documented that DPM incidence is heavily influenced by on farm factors (Lien et al., 2012), thus a low heritability estimate for DPM is not unexpected. The heritability estimate for WB is in line with previous published data (Bailey et al., 2015); this reiterates the importance of understanding the influence of the non-genetic factors on WB. The high residual variance and accompanying low heritability for SB would indicate that non-genetic factors are of significantly greater influence for the variation in expression of this trait; therefore more work is needed to elucidate the risk factors. Compared to the other myopathies, WS had higher heritability estimate of 0.25 which is similar to previous published data where a heritability range of 0.185–0.338 for WS was reported (Bailey et al., 2015). Despite the higher heritability the residual variance still dominates indicating that the non-genetic effects are of greater importance for the variation seen with WS.

The genetic correlations between the traits suggests that genetic selection for increased BW and BY has not had a concomitant increase in the genetic propensity for the expression of breast myopathies. The genetic correlations between the production traits and the BMM are low to moderate indicating that only a small proportion of the genetic component influencing BW and BY is shared with the genetic component of the BMM. Similarly, the phenotypic correlations between the myopathies and production traits are also low to moderate, ranging from 0.02 to 0.23. The lack of strong phenotypic correlations indicates that the myopathies can occur in birds of all sizes with a range of BW and BY. These results indicate that selection for increased BW and BY is not the sole cause of the myopathies. A comprehensive study comparing conventional and slower growing broilers by Muth and Valle Zárate (2017) demonstrated there were no detrimental effects on meat quality in the faster growing broilers. Therefore, understanding why BMM occur requires more extensive research into all factors that can impact upon muscle development and subsequent meat quality.

Within this analysis the contribution of the PEM to the phenotypic variance of each trait was estimated. For this dataset the PEM for BW, BY, WB and WS is negligible when compared to the heritabilities and standard errors for those traits. However, for DPM and SB the PEM was slightly larger and had it been ignored in the analysis the heritability for DPM would have increased from 0.06 to 0.09 and SB would have increased from 0.04 to 0.074. This highlights the importance of fitting the PEM when estimating genetic parameters in chicken datasets that are characterised by families with large sets of full sibs to ensure accurate discrimination of the additive genetic variance.

Despite the modest influence of genetics on the myopathies, the genetic component has not been overlooked by primary breeders as it can be used as part of a holistic approach to reduce the incidence and severity of the myopathies. The results presented here show that, from the perspective of genetic selection, it is possible to continue to improve breast yield and body weight whilst reducing the genetic propensity for the expression of all the BMM as described by Bailey et al. (2015). Since the novel myopathies were first recognised, Aviagen took advantage of the modest genetic component of the three myopathies and incorporated them into a selection strategy. This is the first report showing the impact of this selection strategy and how myopathies can be selected against without impacting upon performance. The comparison of HG broilers with the current commercial broiler empirically shows that the ongoing genetic selection against WB is effective and that it can be done whilst still improving production traits such as BY. One assumption made in the interpretation of the results for study 2 is that the reduction in relative incidence of the myopathies in the HG broilers is due only to genetic selection. It is possible that there are confounding factors which could influence the result especially as the parents of each group of broilers were reared in different environments. However, the PS in both locations were reared under similar management parameters and feed specification to limit these confounding effects. Furthermore, the broiler progeny of the HG and current commercial broilers for each of the four hatches were incubated and subsequently reared together to further limit the impact of environmental factors which could influence the manifestation of WB. In this study there was hatch to hatch variation in bird performance; season is known to impact upon broiler performance (Koknaroglu and Atilgan, 2007; Osti et al., 2017) and as the hatches were placed at different times of the year, season could play a role in the hatch to hatch variation.

**FIGURE 6:**
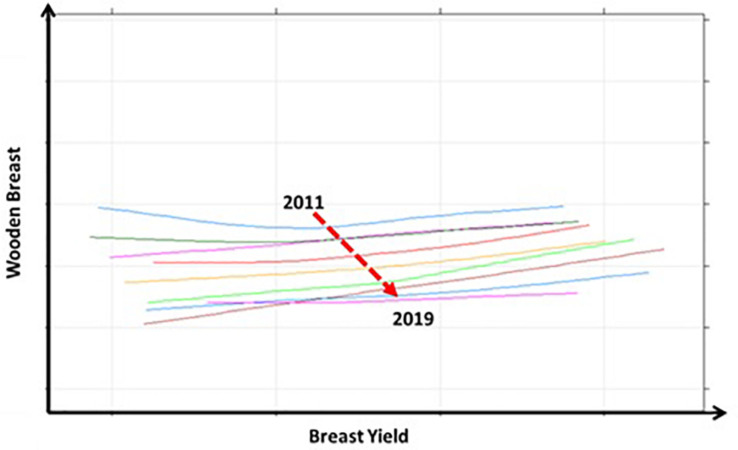
The long term relationship between breast yield (%) and wooden breast (%) for the years 2011 to 2019 is displayed in this graph. The different coloured lines each represent the year long relationship between breeding values for each trait for each year. The broken arrow shows the movement of the mean breeding value for each trait from 2011 to 2019. It shows that there has been a yearly decrease in the mean breeding value for wooden breast whilst the mean breeding value for breast yield has increased.

The ability to select for increased breast yield whilst reducing the genetic propensity for WB is testament to the low genetic correlation between the two traits. The effect of this selection over time can be seen in Figure 6 which shows the relationship between BY % and WB % between 2011 and 2019; each line in this graph represents the relationship between the breeding values for each trait within each year. The broken arrow demonstrates the joint direction of the average breeding value for each trait involved in the trade off. Although it has often been suggested that the only viable options to reduce BMM is to either reduce genetic selection for BW and BY or use slower growing alternatives, here we show that meat quality can be improved whilst improving performance as part of a balanced multi-trait breeding goal.

Whilst the genetic component of the myopathies can be targeted through selection, it must be acknowledged that genetic selection is not the sole solution for reducing the incidence of the myopathies. Again, non-genetic effects have a much greater influence on the variation in BMM and therefore offer a more successful short term opportunity to significantly reduce their incidence. Genetic selection against the myopathies is more of a long term strategy owing to the low heritabilities of the BMM and the time taken for genetic progress to pass from the elite population to the commercial broiler. Ultimately, the successful reduction of myopathies in broiler chickens will be the result of a holistic approach to understand and account for all the influencing factors.

Flock growth and performance are influenced by a wide range of environmental and management factors such as brooding, nutrition, temperature and ventilation. Understanding how these factors influence bird growth in terms of muscle development may give more insight into the pathophysiology of the myopathies. Muscle growth, development and maintenance are reliant on the satellite cells; these are multipotent stem cells located on the periphery of muscle fibres and provide the required myogenic precursors for muscle fibre growth and repair (Moss and Leblond, 1970). Incubation, brooding and early chick management have all been shown to influence satellite cells and play a role in muscle growth and development thus influencing final breast yield and composition of the breast muscle. For example, thermal manipulations during incubation have been shown to increase myoblast activity leading to increased satellite cell number and increased breast yields in the broiler (Piestun et al., 2009, 2011, 2013; Al-Musawi et al., 2012). Satellite cells are extremely active post hatch and proliferate to increase their population within the muscle, however, as the bird gets older the activity of these cells decrease which could have an impact on their ability to support the muscle later in life (Mann et al., 2011; Harthan et al., 2013; Daughtry et al., 2017). Failure to provide newly hatched chicks with adequate nutrition during brooding can result in reduced satellite cell activity and number (Harthan et al., 2014; Powell et al., 2014). There can be long term consequences of impaired satellite cell function and development with regards to meat quality in the older broiler. Satellite cells in birds which are feed restricted during early life and then have full access to *ad lib* have activity associated with muscle fibre degeneration and adipogenesis (Velleman et al., 2010) leading to lower protein and higher fat in breast tissue (Zhan et al., 2007). Heat stress during the early life of the chick has been demonstrated to have a negative impact on satellite cell function leading to increased collagen and fat deposition in breast muscle (Piestun et al., 2017; Patael et al., 2019). Thus it is very important to ensure incubation, farm management and feed availability are optimal to encourage early chick growth to promote maximal satellite cell proliferation and activity for supporting muscle development. There have been a number of technical articles from industry (Aviagen Ltd, 2019) and peer reviewed articles highlighting potential nutritional and management related strategies to reduce the myopathies whilst maintaining optimal performance. Nutritional strategies include modifying the broiler growth curve through lysine reduction (Meloche et al., 2018a, b) or increasing phytase levels in the feed to give support to the muscle (Greene et al., 2019). Management and nutritional strategies which successfully reduce BMM incidence provide a great opportunity to further understand the key biological pathways identified by gene expression, metabolomic and proteomic analyses of breast tissue affected by myopathies. This could help distinguish cause and effect when it comes to characterising the underlying aetiology of each of the conditions. The BMM can appear together or individually likely indicating they are separate conditions, however, the biological pathway studies reveal similar key pathways for each BMM.; here we show low phenotypic correlations between the BMM ranging from -0.02 to 0.25, and the genetic correlations are low to high ranging from -0.04 to 0.74. It’s probable that there is a common origin to the BMM and more research is needed to untangle which factors influence the manifestation of a particular BMM; this may depend upon what the initial trigger is, the age of the bird when it occurs or even the sex of the bird.

The aim of this paper was to characterise the genetic basis of BMM found in broiler chickens and their relationship with body weight and breast yield. Data from a pedigree broiler pure line showed that the residual variance accounted for a greater proportion of the phenotypic variance of the myopathies than the genetic variance. This indicates that the non-genetic factors have a greater impact on the variance of the myopathy traits than the genetic factors. It has been previously described how balanced breeding goals can lead to cumulative improvements in traits that have low genetic basis such as the myopathies (Neeteson, 2010; Kapell et al., 2012a, b; Bailey et al., 2015). Using a HG broiler and the current commercially available broiler we demonstrate the effectiveness of the balanced breeding approach; the results show that BY can be successfully improved whilst at the same time reducing the genetic component of WB. In the long term genetic selection will help reduce the incidence of all myopathies, however, in the shorter term exploring management strategies to capture the non-genetic effects is critical.

## Data Availability Statement

The datasets presented in this article are not readily available because it contains propriety information. Access to the data will be permitted by agreement. Requests to access the datasets should be directed to RB, rbailey@aviagen.com.

## Ethics Statement

The data used in this study came from the routine data collection and operation of the Aviagen breeding program where all animals are under the care of the Aviagen Veterinary Department, Huntsville, AL, United States. The data used in this trial is collected from birds going to slaughter as part of normal commercial poultry processing through an abbatoir. These birds were not experimental animals and there were no experimental procedures carried out on them.

## Author Contributions

All authors were involved with the conception of the research. RB drafted the manuscript which was revised and reviewed by SA and ES.

## Conflict of Interest

All authors are employed by Aviagen Ltd.

## Footnotes

1 www.aviagen.com

## Abbreviations

BMM: Breast muscle myopathies
BW: Body weight
BY: Breast yield
DPM: Deep pectoral myopathy
GGP: Great grandparent
GP: Grandparent
HG: High generation
PS: Parent stock
SB: Spaghetti breast
WB: Wooden breast
WS: White striping.
